# The Use Patterns of Medicaid Home and Community Based Services Among Medicare/Medicaid Beneficiaries With Dementia

**DOI:** 10.3389/fpubh.2021.708402

**Published:** 2021-10-28

**Authors:** Di Yan, Sijiu Wang, Helena Temkin-Greener, Shubing Cai

**Affiliations:** ^1^School of Medicine and Dentistry, University of Rochester, Rochester, NY, United States; ^2^Division of Biological Sciences, Department of Public Health Sciences, University of Chicago, Chicago, IL, United States

**Keywords:** Medicaid home and community based services, Alzheimer's disease and related dementias, Medicare-Medicaid dual eligible beneficiary, Medicaid policy, race, neighborhood socioeconomic status

## Introduction

Older adults with Alzheimer's disease and related dementias (ADRD) often have high care needs (1). As their care needs increase, many are placed in nursing homes (NHs), even if they would prefer to live in the community (2). The Medicaid home and community-based services (HCBS) program provides health and supportive services that may allow Medicaid beneficiaries with cognitive and physical impairments to maintain living in the community, delaying or preventing NH placement (3). The HCBS cover services such as durable medical equipment, transportation, hospice care, residential care, personal care, home health, and other waiver services (4). As older adults with ADRD have different care needs than other populations, their utilization patterns of these services can be unique. However, to date, the extent to which these services are differentially used to help maintain community living among older adults with ADRD is not known. Furthermore, while prior studies have noted the existence of racial differences in care needs and individual preferences among persons with ADRD (5, 6), it is unknown whether differences in HCBS utilization patterns between Black and white service users with ADRD also exist. Lastly, it is unknown whether the patterns of HCBS utilization vary by the socio-economic status of the community, which can be closely related to racial differences in individual health, care needs, and ability to maintain community living.

Therefore, the main objective of this study is to address these gaps in knowledge. More specifically, we explored the differences in the pattern of Medicaid HCBS utilization among Black and white Medicare-Medicaid dual eligible older adults with ADRD, and how such racial differences varied by the socio-economic status of the community in which an individual resides. This is an important question to address in order to identify services that may be potentially under-utilized, so as to better target services to the needs of the population.

## Materials and Methods

### Data

The Medicaid Analytic eXtract (MAX) Personal Summary (PS) and Other Therapy (OT) files were obtained for all eligible individuals in the U.S. between 2010 and 2012, and individuals in 28 states in 2013 (due to the availability of data at the time of the data request). These data were then linked with the following 2010–2013 dataset at the individual level: Minimum Data Set (MDS) 2.0/3.0, Medicare Master Beneficiary Summary File (MBSF), and Medicare Provider Analysis and Review (MedPAR). The MAX PS and OT files include the utilization and expenditure of all types of HCBS for each Medicaid enrollee. The MDS contains information on individuals' NH placements for those admitted to Medicaid- and/or Medicare-certified NHs. MBSF contains information on individuals' demographics, chronic conditions, and death date information for Medicare beneficiaries. The MedPAR file contains information on hospitalization events for Medicare beneficiaries.

### Study Population

We initially included 1,758,640 new HCBS users with ADRD who were dually eligible for Medicare and Medicaid and started to use HCBS between February the 1st 2010 through January the 1st 2013. Medicare and Medicaid dually eligible fee-for-service (FFS) beneficiaries were identified using MBSF and MAX PS files. We excluded the Medicare/Medicaid managed care enrollees because their HCBS utilization and hospitalization data were not available (8.6% of the sample was excluded). Diagnosis of ADRD was based on the MBSF chronic condition files. The diagnosis of ADRD in the MBSF chronic condition file was determined based on the ICD-9 codes of all Medicare claims within the past 3 years (7). New HCBS users were defined as those who did not have HCBS episodes in the prior 30 days, based on the OT records. If an individual had multiple eligible episodes over the study period, we only selected the first HCBS use. In total, the final analytical sample included 1,164,225 individuals.

### Variables

The outcome variables included the utilization of each of the following HCBS service categories within 365 days of the first state date of HCBS (i.e., the follow-up period): durable medical equipment, transportation, hospice care, residential care, personal care, home health, targeted case management, adult day care, private duty nursing, and other waiver services. Each service type was defined as dichotomous, indicating whether such services had been used or not during the follow-up period. These service categories were identified based on MAX OT file Community Based Long-term Care (CLTC) flag and included both the state plan services and the waiver services (4). These services account for more than 95% of Medicaid HCBS spending.

Key variables of interest were race and neighborhood socioeconomic status. The race of beneficiaries was dichotomized as white or Black using the MBSF Research Triangle Institute (RTI) race variable (8). Neighborhood socioeconomic status was determined based on the 2015 Area Deprivation Index (ADI) (9). The ADI is a validated, neighborhood-level composite index reflecting 17 social determinants of health such as income, education, employment, and housing quality. Its rankings range from 1 to 100, with more disadvantaged neighborhood conditions designated by a higher score. We dichotomously defined disadvantaged neighborhoods as those with an ADI score >55, which was the average ADI score of the study sample.

We also included several covariates, including age, gender, years since first diagnosis of ADRD, chronic conditions (e.g., diabetes, depression, cardiovascular disease, cancer, etc.), and county-level HCBS intensity defined as Medicaid spending on HCBS per user per month.

### Analysis

All analyses were conducted at the individual level. We firstly fit a set of linear probability models with county fixed-effect and robust standard errors to examine the relationship between race and the use of each type of HCBS without accounting for other covariates. The linear probability model approximates the logit model and provides the direct interpretation of the coefficients (i.e., change in the probability of outcomes given one unit change in an independent variable) (10, 11). In these models, the coefficients of the race captured overall racial differences in the probability of using each type of HCBS between Black patients and white patients. We then estimated a set of models by adding additional individual characteristics (e.g., age, gender, diagnosis years of ADRD, chronic conditions) and county-level HCBS intensity to explore how much of the overall racial differences could be explained by these variables. Lastly, we stratified the analyses by the socio-economic status of the neighborhood (i.e., whether a community was economically disadvantaged or not, based on ADI score) and examined whether the racial difference in the pattern of HCBS utilization varied with these two types of neighborhoods (12). Lastly, although not included in the main analyses, we also compared the pattern of other health care utilization, including hospitalization (based on MedPAR data) and nursing home placement (based on the MDS data), and any mortality within 365 days of HCBS initiation date.

All analyses were performed using SAS 9.4 (SAS Institute Inc.) and STATA 16 (StataCorp LLC. College Station, TX). This study has been reviewed and approved by the Research Subjects Review Board. The final dataset was saved in SMDNAS College-based data storage. All authors have no conflicts of interest.

## Results

Among the analytical sample, 79% of care recipients were white and 21% were Black. The annual spending on HCBS services was higher among white beneficiaries than among black beneficiaries ($5,939 vs. $5,163, *P* < 0.01). Table 1 compares the pattern of HCBS use and individual characteristics by race. Overall, hospice care had the highest median spending ($27,622 per user per year), followed by residential care ($14,695 per user per year) and personal care ($5,241 per user per year). Personal care had the longest median duration days among HCBS users (152 days per user per year) followed by other waiver services (82 days per user per year) and home health (25 days per user per year). In addition, Black recipients were generally younger, but the distribution of chronic conditions was mixed–for example, Black patients were more likely to have chronic kidney disease, stroke, and diabetes, but were less likely to have depression, anxiety disorders, and osteoporosis than their white counterparts. White HCBS users were also more likely to have NH placements than were Black users. Among those users without any NH placement within 1 year of HCBS use, Black users had a higher hospitalization rate but a lower mortality rate than white users (more details shown in Table 2).

**Table 1:** Distribution of HCBS use and individual characteristics by race.

	**Race**		
	**White**	**Black**	**All**
**Number of individuals** (%)	916,422 (78.72%)	247,803 (21.28%)	1,164,225 (100.00%)
**The penetration of each HCBS type utilization**			
Medical equipment	58.58	65.37	60.03
Transportation	40.07	43.39	40.78
Hospice care	14.42	7.61	12.97
Other waiver service	10.83	13.90	11.49
Residential care	6.15	2.16	5.30
Personal care	7.70	10.74	8.35
Home health	5.92	6.29	6.00
**Median annual spending on each HCBS type**			
Medical equipment	$128	$134	$129
Transportation	$190	$187	$190
Hospice care	$27,827	$26,330	$27,622
Other waiver service	$2,570	$2,875	$2,632
Residential care	$14,747	$13,792	$14,695
Personal care	$5,104	$6,338	$5,241
Home health	$1,588	$2,176	$1,745
**Median duration days of each HCBS type within 1 year**			
Medical equipment	2	3	2
Transportation	2	3	2
Hospice care	11	10	11
Other waiver service	83	79	82
Residential care	13	13	13
Personal care	152	156	152
Home health	25	33	25
Number of individuals	916,422	247,803	1,164,225
**Individual factors**			
Age	77.98	75.10	77.36
(SD)	(13.06)	(13.27)	(13.16)
Years since ADRD diagnosis	2.07	1.66	2.03
(SD)	(1.90)	(1.70)	(1.67)
Average spending on HCBS within 1 year	5938.67	5163.35	5772.75
(SD)	(15452.27)	(12281.15)	(14834.19)
**Average monthly spending per HCBS user at county level**			
< $700	30.82	30.86	30.83
≥$700 and <1,000	37.44	35.94	37.12
≥$1,000	31.74	33.20	32.05
Female	30.59	34.10	31.34
Living in disadvantaged neighborhoods	48.50	32.70	45.15
Acute myocardial infarction	8.13	6.31	7.74
Chronic kidney disease	36.27	44.27	37.98
Chronic obstructive pulmonary disease	45.22	36.45	43.34
Heart failure	52.93	53.88	53.14
Diabetes	48.12	59.57	50.57
Ischemic heart disease	64.14	62.46	63.78
Depression	64.79	48.18	61.23
Osteoporosis	32.17	16.14	28.74
Rheumatoid arthritis/osteoarthritis	63.89	59.66	62.98
Stroke/transient ischemic attack	33.38	37.84	34.33
Asthma	17.96	18.11	17.99
Cancer	14.07	13.64	13.98
Anxiety disorders	37.56	22.29	34.29
Bipolar	13.16	9.53	12.38
Obesity	16.87	19.72	17.48
Death rate within 1 year	26.69	19.69	25.20

*The differences among the two race groups of all variables were statistically significant at 0.01 level*.

**Table 2:** Nursing home placement, hospitalization, and mortality rate within 1 year among HCBS users by race.

	**Race**			
	**White**		**Black**	
	***N***	**%**	***N***	**%**
Any nursing home entry	604,162	65.9%	133,825	54.0%
Community stayer without nursing home entry	312,260	34.1%	113,978	46.0%
Community stayer with hospitalization	87,576	28.0%	36,523	32.0%
Community stayer mortality rate	41,477	13.3%	11,883	10.4%
All HCBS user	916,422	100%	247,803	100%

*The differences in the nursing home placement, hospitalization, and mortality rate between blacks and whites were statistically significant at 0.01 level*.

**Figure 1:**
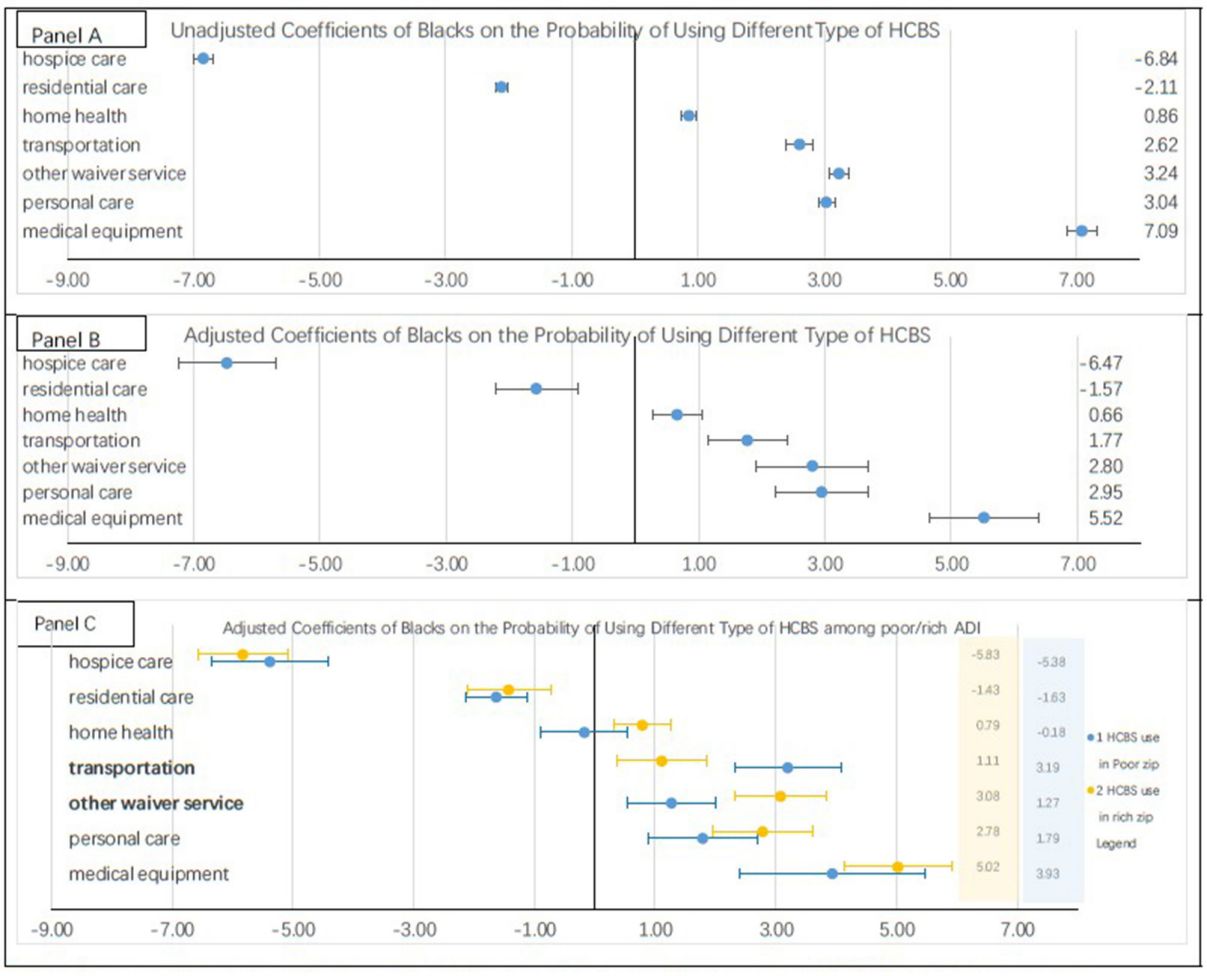
Users' probability of using each type of HCBS. **(A)** shows the results of linear probability regressions on different types of HCBS. The values are the coefficients of blacks on the probability of using different types of HCBS. The regression models added county fixed effect but did not control other variables. **(B)** shows the results of linear probability regressions on different types of HCBS. The values are the coefficients of blacks on the probability of using different types of HCBS. The regressions adjusted individual characteristics (i.e., age, gender, and diagnosis years of ADRD), chronic conditions (e.g., diabetes, depression, cardiovascular disease, cancer etc.), county level Medicaid spending on HCBS per HCBS user per month. **(C)** shows the results of linear probability regressions on different types of HCBS among individuals in disadvantaged neighborhoods and non-disadvantaged neighborhoods, respectively. These regressions adjusted covariates included in the **(B)**. Rich communities are those with 2015 ADI <55. We use 55 as a cutoff point because the mean ADI of the study population is 55.3. With this cutoff 55% of sample was in rich communities.

Figure 1A shows the unadjusted probabilities (i.e., without controlling for other covariates) of using each type of HCBS among Black beneficiaries vs. white ones. Being Black was associated with a 6.8 percentage point lower probability of using hospice care compared to white individuals (*P* < 0.01), and a 2.1 percentage point lower probability of using residential care, compared with whites (*P* < 0.01). In contrast, Black care recipients had a higher probability of using the medical equipment (5.5 percentage point, *P* < 0.01), personal care (3.0 percentage point, *P* < 0.01), other waiver services (3.2 percentage point, *P* < 0.01), transportation (2.6 percentage point, *P* < 0.01), and home health provisions (0.9 percentage point, *P* < 0.01) than white service recipients with ADRD.

Figure 1B presents the adjusted probability of using each type of HCBS among Black patients vs. white patients after controlling for individual-level covariates and county-level HCBS intensity. The findings were similar to those in unadjusted models: Black patients had a lower probability of using hospice care and residential care, but a higher probability of using the other five types of HCBS than white patients did. The racial differences in using medical equipment (5.5 vs. 7.1 percentage points, *P* < 0.05) and transportation (1.8 vs. 2.6 percentage points, *P* < 0.05) were smaller in adjusted models than those in unadjusted models.

Figure 1C illustrates racial differences in the adjusted probabilities of using each type of HCBS among those residing in disadvantaged vs. non-disadvantaged neighborhoods, respectively. In both of the stratified samples, Black residents had a lower probability of using hospice care and residential care than did white residents. In addition, Black service users had a higher probability of using medical equipment, personal care, other waiver services, and transportation than did white users. The difference in using transportation between Black and white patients was larger in disadvantaged neighborhoods than that in non-disadvantaged neighborhoods (3.2 vs. 1.1 percentage points, *P* < 0.05). In contrast, the racial difference in using other waiver services was smaller in disadvantaged neighborhoods than that in non-disadvantaged neighborhoods (1.3 vs. 3.1 percentage point, *P* < 0.05).

## Discussion

In this study, we examined racial differences in the patterns of HCBS use among Medicare-Medicaid duals with ADRD. We found that the total spending on HCBS services was lower among Black enrolees than that among white enrolees. Black patients with ADRD appeared to use different types of services than their white counterparts. Such differences could not be fully explained by the selected sets of individual characteristics. Moreover, HCBS use patterns varied by the socio-economic status of the community in which an individual resides.

While we found that Black patients tended to use certain services more than white patients did, it is unclear what may have driven such racial differences. One possible explanation may be related to different care needs between Black and white HCBS users with ADRD. Black HCBS users were generally younger, had fewer years with an ADRD diagnosis, and were less likely to approach the end of life than white users. Thus, they may be more likely to rely on less expensive HCBS (such as transportation and medical equipment) to support their community living than white users. Moreover, the different distribution of comorbidities between white and Black people with ADRD may also contribute to the different patterns of HCBS utilization. For example, Black ADRD patients were more likely to have chronic kidney disease and stroke than white ADRD patients. Individuals with these comorbidities always need transportation to and from the hemodialysis center or often depend on emergency care (13, 14). Therefore, individuals with these comorbidities may be more likely to use Medicaid transportation services. Indeed, after controlling for individual health conditions, the racial differences in using transportation services were reduced.

The findings of this study further suggest that neighborhood socioeconomic status may also influence the pattern of HCBS utilization. For example, Black individuals residing in disadvantaged neighborhoods had a higher likelihood to use Medicaid transportation assistance than Black individuals in non-disadvantaged neighborhoods. This might be related to the availability of relevant medical services in these communities. Studies have suggested that individuals in more disadvantaged neighborhoods may have to drive further to receive specialist care (15) thus leading to higher utilization of transportation services.

Lastly, the detected racial differences in HCBS utilization may also be related to individual preferences with regard to different services. For example, our findings indicate that Black patients are less likely to enroll in hospice than white patients, and such racial differences do not seem to be affected by the set of observed individual characteristics. It is likely that Black service users prefer more intensive treatment due to the historical undertreatment of black patients (16). The Medicaid hospice role is very small among Medicare and Medicaid dually eligible beneficiaries (Medicare and Medicaid accounted for about 74 and 7% of total hospice revenues, respectively) since the Medicaid programs just pay Medicare hospice copayments or some optional service that are not covered by Medicare (17). The total hospice care use may be different from that found in this study.

Several limitations should be mentioned. First, although the study population in this study are Medicare-Medicaid dually eligible, we were not able to examine whether they had barriers, other than insurance status, in access to different types of HCBS services. Secondly, some potential drivers of HCBS use, such as for example personal preferences or the availability of family caregiver support, cannot be ascertained from claims data. Thirdly, this study just included fee-for-service beneficiaries but excluded beneficiaries covered by Managed Care. Therefore, the results of this study may not be applied to those Managed Care beneficiaries. Future research should further examine other potential reasons underlying the difference in HBCS use. In addition, this study did not include Medicare-covered services. However, Medicare and Medicaid have different roles in paying for health services. For example, Medicare home health services are used for post-acute rehabilitative care needs, while Medicaid reimburses for other in-home personal attendant services that are specifically excluded from Medicare coverage (18, 19). Home health services paid for by Medicare and those paid for by Medicaid are different and are not substitutes for each other. Thus, we do not think that focusing on Medicaid HCBS services alone is a limitation. Lastly, our identification of ADRD is based on the Medicare data. Although it is possible that we under-identify the population with ADRD, this is the best data source available to us to identify ADRD population. Despite these limitations, to the best of our knowledge, this is the first study that used national data to examine racial differences in HCBS use patterns and health outcomes among HCBS users with ADRD.

The findings of this study shed light on how HCBS services are used by white and Black duals with ADRD. These HCBS use patterns differences among white and black individuals with ADRD could be important as policymakers target service availability to this population to improve care and delay or prevent institutional care.

## Data Availability Statement

According to the CMS requirement of MAX data, we cannot open the raw data of this study to the public. Instead, the raw data will be saved in the College-based data storage according to CMS and University of Rochester Data use policy.

## Author Contributions

DY, SW, HT-G, and SC contributed to conception and design of the study. DY and SW organized the database. DY performed the statistical analysis and wrote the first draft of the manuscript. All authors contributed to manuscript revision, read, and approved the submitted version.

## Conflict of Interest

The authors declare that the research was conducted in the absence of any commercial or financial relationships that could be construed as a potential conflict of interest.

## Publisher's Note

All claims expressed in this article are solely those of the authors and do not necessarily represent those of their affiliated organizations, or those of the publisher, the editors and the reviewers. Any product that may be evaluated in this article, or claim that may be made by its manufacturer, is not guaranteed or endorsed by the publisher.
